# Editorial: Weight-related behaviors and outcomes in children and youth with intellectual and developmental disabilities

**DOI:** 10.3389/fped.2023.1295630

**Published:** 2023-10-06

**Authors:** Aviva Must, Carol Curtin, April Bowling, Sarabeth Broder-Fingert, Linda G. Bandini

**Affiliations:** ^1^ School of Medicine, Tufts University, Boston, MA, United States; ^2^ Eunice Kennedy Shriver Center, University of Massachusetts Chan Medical School, Worcester, MA, United States; ^3^ School of Nursing and Health Sciences, Merrimack College, Andover, MA, United States; ^4^ Department of Psychiatry, University of Massachusetts Chan Medical School, Worcester, MA, United States; ^5^ Department of Pediatrics, University of Massachusetts Chan Medical School, Worcester, MA, United States

**Keywords:** autism spectrum disorder, eating patterns, screen time, physical activity, intellectual and developmental disabilities, sedentary behavior

**Editorial on the Research Topic** Weight-related behaviors and outcomes in children and youth with intellectual and developmental disabilities

Poor diet, low physical activity levels, and high levels of sedentary behavior are associated with obesity and a host of other chronic diseases, including diabetes, coronary heart disease, hypertension, stroke, obstructive sleep apnoea, and many cancers. Childhood obesity rates in the general population have grown rapidly, increasing almost five-fold over the last several decades (1). Studies from around the world also demonstrate that children with intellectual and developmental disabilities (IDD) are at higher risk for obesity than those without IDD (2, 3).

Investigations in typically developing children suggest that changes in obesity prevalence have been fueled by changes in eating patterns, physical activity engagement, and sedentary behaviors. Although children with IDD are exposed to the same obesogenic environments as their typically developing counterparts, they appear to experience additional risk factors associated with their disability and structural disability-related barriers to a healthy lifestyle. These include food selectivity, medication use, feeding problems, behavioral challenges, body composition alterations, and individual- and community-level barriers to physical activity (4). Notably, the 2023 American Academy of Pediatrics Clinical Obesity Guideline emphasized the importance of screening and treatment for obesity in the IDD population (5).

This Research Topic was curated by the Healthy Weight Research Network (HWRN) for Children with Autism Spectrum Disorder and Developmental Disabilities, an interdisciplinary research network funded by the Maternal Child Health Bureau within the U.S. Health Resources & Services Administration (6). The mission of the HWRN is to promote the development of evidence-based solutions to achieve healthy weight in children with autism and other IDD, and to disseminate research findings to broad and diverse audiences. This diverse set of papers reflects the wide range of research questions, study designs, and disciplinary perspectives that characterize this important research area and adds to our understanding of weight-related behaviors in children with IDD. The papers in this Research Topic focus primarily on autistic children and children with Down syndrome.

Parents of children with IDD often experience challenges around eating and leisure time behaviors of their children beyond those of parents of children without IDD. Within this Research Topic we share three original articles on different aspects of parenting children with IDD. Magaña et al. describe associations of overweight/obesity status of Latino children with IDD with parenting practices around food and physical activity based on interviews with parent-child dyads. They found that greater parental use of controlling dietary strategies was associated with *lower* BMI percentile. A qualitative study by Blaine et al. contrasts parent and interdisciplinary health professionals' perspectives on priorities, barriers, and facilitators to nutrition-related care for autistic children. Among the findings from their thematic analysis was that parents tended to emphasize the importance of addressing food selectivity, behavioral eating challenges, sensory issues, and sleep disturbances affecting appetite. Caldwell et al. leveraged a triangulated qualitative approach to explore facilitators and barriers to healthy behaviors among young children (12–36 months old) with Down syndrome. Their in-depth reflexive thematic analysis revealed child-level facilitators (high activity and sound sleep) and barriers (co-occurring conditions and eating behaviors) as well as family and community factors that included role modelling, time constraints, and social support.

Parents and clinicians are eager for access to effective interventions to promote healthy weight-related behaviors. Our Research Topic includes four contributions that reflect innovative adaptations to meet the needs of children with IDD and their families. Ptomey et al. report a post-hoc secondary analysis of a successful randomized 18-month weight management trial to assess whether outcomes differed between adolescents with Down syndrome and adolescents with other IDDs. Study authors found no significant differences in weight loss or compliance with intervention elements post randomization. The remaining three investigations were conducted among participants with autism. Kral et al. assessed the initial efficacy of an mHealth nutrition intervention to encourage healthy foods and discourage less healthy choices over 3 months among autistic children aged 6–10 using a randomized design with a waitlist control. Initial findings suggested only participants who initially consumed few fruits and vegetables and were highly engaged with technology increased intake over 3 months. Remote delivery was also utilized in a 4-week beta-test of a single-arm exergaming intervention that adapted an existing approach. Hatfield et al. report high feasibility, acceptability, and engagement with the progressive exergame schedule, Fitbit step-tracking, health tip and exercise videos—supported with weekly telehealth coaching. Lastly, Atkins et al. conducted a qualitative study to inform the development of a family-based intervention that centers the family dog as a vehicle for weight-related behavior change in autistic children. Interviews with parent-child dyads illuminated the strong relationship between the child and the pet dog and the dog's active role in family life; challenges related to physical activity and nutrition; and positive views on potential intervention strategies.

This Research Topic also features the results of three original observational epidemiological studies that leverage population-based datasets employing an open-science model. Because the IDD population is relatively small, population-based studies of IDD require very large samples to yield adequate numbers of individuals with IDD for adequately powered investigations. Harris et al. identified a strong association between autism symptoms and eating problems at the between-person level, but little evidence for consistent longitudinal effects at the within-person level using five waves of childhood data gathered in the Generation R Study, conducted in the Netherlands. Two studies used baseline data from the Adolescent Brain and Cognition Development Study, a population-based longitudinal study conducted in the U.S. In their exploration of racial and ethnic obesity disparities and the potential mediating contributions of select social determinants of health among autistic boys, Magaña et al. found significant differences in some social determinants; only food insecurity mediated differences between Black and White children. A second cross-sectional analysis by Must et al. identified important differences in the screen time habits (passive screen time, videogame playing, and social screen time) between male and female pre-adolescents with and without autism, but their associations with obesity were similar in the two groups.

It is essential to assess body composition and energy expenditure in individuals with Down syndrome in order to determine energy needs and thereby prevent excess weight gain. In this Research Topic, Polfuss et al. share their innovative protocol to gather energy expenditure data using doubly-labeled water from youth with Down syndrome, using video cameras to monitor data collection remotely.

There is a recognized need for practice guidelines for the treatment and management of obesity in children and adolescents with IDD (ref AAP). Ptomey et al. assembled a workgroup to develop such guidelines that incorporate the specific physiological and cognitive needs of youth with Down syndrome with obesity. An expert panel then reviewed each recommendation and rated its strength and the strength of the evidence, resulting in eight recommendations available for clinicians to use with patients and their families.

Together, the studies that comprise this Research Topic contribute to the evidence base of well-designed studies and highlight pioneering approaches to research that seeks to promote healthy weight-related behaviors for children with IDD for their long-term health and well-being. With much investigation and translation to practice yet to be done, we hope this collection serves to inspire novel research questions, innovative approaches to implementation, and attract new investigators from a range of disciplines and training backgrounds.
